# PRV Induces Neurological Inflammatory Injury by Activating Necroptosis of Brain Tissue

**DOI:** 10.3390/microorganisms13071531

**Published:** 2025-06-30

**Authors:** Chunzi Peng, Jinwu Zhang, Changxu Wu, Danning Liu, Jing Liang, Maojie Lv, Shisen Yang, Xiaoning Li, Yingyi Wei, Hailan Chen, Jiakang He, Tingjun Hu, Meiling Yu

**Affiliations:** Guangxi Key Laboratory of Animal Breeding, Disease Control and Prevention, College of Animal Science and Technology, Guangxi University, Nanning 530004, China

**Keywords:** PRV infection, neurological inflammatory injury, necroptosis, NF-κB signaling pathway

## Abstract

Pseudorabies virus (PRV) can infect a wide range of animal species, including swine and rodents. Infection in pigs is associated with significant economic losses in the global pork industry and is characterized by acute, often fatal disease, as well as central nervous system (CNS) invasion, which leads to neurological manifestations. Although PRV replication has been extensively characterized in certain murine neuronal cell lines such as Neuro-2a, the mechanisms underlying PRV-induced neuroinflammatory injury and necroptosis remain largely unclear. In this study, Kunming mice and mouse astrocytes (C8-D1A) were infected with PRV-GXLB-2013 at different doses to evaluate neurological injury and inflammatory responses. Given that the NF-κB/MLKL signaling pathway was found to be activated during PRV infection, a selective MLKL inhibitor, necrosulfonamide (NSA), was applied to investigate the role of necroptosis in PRV-induced neuroinflammatory damage. Mice infected with higher viral doses showed increased mortality, severe neurological symptoms, elevated brain inflammation, and pathological changes. In C8-D1A cells, PRV infection significantly upregulated inflammatory cytokines and key components of the NF-κB/MLKL pathway. Importantly, NSA treatment markedly reduced these inflammatory responses, mitochondrial damage, and cellular necrosis. Collectively, these findings suggest that PRV infection triggers neuroinflammatory injury through the activation of necroptosis and the NF-κB/MLKL signaling pathway. This study provides novel mechanistic insights into PRV-induced neurological damage and highlights potential therapeutic targets for intervention.

## 1. Introduction

PRV, also known as Aujeszky’s disease virus, is a highly contagious swine pathogen that spreads rapidly via direct contact or fomite transmission, causing severe respiratory, reproductive, and neurological disorders in pigs, leading to substantial economic losses globally [1,2]. In addition to swine, PRV exhibits broad host tropism and can infect ruminants, carnivores, rodents, and other mammals. In these non-reservoir hosts, PRV typically results in fatal encephalomyelitis with pronounced neurological deficits, such as intense pruritus, motor incoordination, and ataxia, indicative of peripheral and CNS dysfunction [3]. Notably, outbreaks in domestic and wild carnivores, particularly dogs, have been increasingly reported, often linked to the consumption of infected pork or contact with infected pigs. Affected dogs exhibit severe neurological signs and high mortality rates, emphasizing their susceptibility to PRV infection [4]. Emerging evidence also highlights an increasing incidence of human PRV infections, which are associated with acute encephalitis, endophthalmitis, and retinal vasculitis, underscoring its growing threat to public health [5,6,7].

PRV, an alphaherpesvirus with a double-stranded DNA genome, belongs to the Varicellovirus genus within the subfamily Alphaherpesvirinae of the Herpesviridae family. It exhibits pronounced neurotropism similar to other members of the Varicellovirus genus. While PRV is not typically considered a classical zoonotic agent, emerging reports suggest that it may pose a potential risk to immunocompromised individuals under specific circumstances. The virus can invade the host nervous system through synaptic connections and retrograde axonal transport mechanisms [8]. PRV enters host cells via envelope glycoproteins that facilitate membrane fusion. Following invasion of the CNS, the virus replicates within neurons and subsequently releases the virus, culminating in severe neuroinflammatory pathogenesis. The neuropathological manifestations typically include neuronal degeneration, reactive astrogliosis, and elevated production of proinflammatory cytokines [9].

Numerous studies have highlighted the relationship that PRV has with different signaling pathways, explaining potential mechanisms of action of the virus. Studies have shown that PRV can activate the NF-κB signaling pathway, and PRV infection induces DNA damage within a short period, leading to ATM activation (ataxia telangiectasia-mutated). Activated ATM further activates IKK kinase (inhibitor of kappa B kinase), which contributes to the phosphorylation and ubiquitination of nuclear factor κB inhibitory factor (IκBα), ultimately leading to IκBα degradation. Subsequently, NF-κB p65 is activated and translocated into the nucleus, where it binds to the target gene’s promoter and regulates the transcription of downstream genes. In addition, PRV inhibits the transcription of IκBα and promotes its proteasome-dependent degradation, thereby blocking the negative feedback regulation of the NF-κB signaling pathway, leading to the continuous activation of the pathway [10,11,12]. Studies have demonstrated that PRV activates the NF-κB signaling pathway, upregulating the expression of TNF-α, a pleiotropic cytokine that critically modulates immune responses, inflammatory processes, and apoptotic pathways [13]. Notably, proinflammatory mediators such as TNF-α trigger the activation of receptor-interacting protein kinase 1 (RIPK1) and RIPK3, which subsequently phosphorylate the downstream effector MLKL. This phosphorylation event culminates in plasma membrane disruption [14], driving RIPK3-dependent necroptosis. Necroptosis is a regulated form of programmed cell death mediated by a signaling cascade involving RIPK1, RIPK3, and MLKL. This cell death pathway is critical in multiple pathological processes, including infection, inflammatory disorders, and, more recently, neurological diseases [15,16,17]. MLKL is the terminal effector within the necroptotic pathway, directly executing plasma membrane rupture and cell death. Given its position downstream of RIPK1/RIPK3 and its specific role in necroptosis (unlike the pleiotropic functions of upstream kinases), MLKL represents a promising therapeutic target with potentially greater specificity than other pathway components [18].

During viral infection, certain viruses can activate the necroptotic pathway to facilitate cytokine release, thereby exacerbating tissue damage and amplifying inflammatory responses [19]. Notably, herpes simplex virus (HSV), a member of the α-herpesvirus subfamily, has been demonstrated to induce necroptosis [20]. However, whether PRV infection triggers neuroinflammation through necroptosis remains unexplored.

In this study, the neuroinflammatory injury induced by PRV-GXLB-2013 infection was investigated in Kunming mice and mouse astrocyte cells (C8-D1A). NSA intervention studies were conducted to investigate the mechanistic basis of PRV infection. The study preliminarily showed that PRV might induce inflammatory injury in the nervous system by activating necroptosis, which provides an important theoretical basis for elucidating the molecular mechanism of inflammatory injury in the nervous system caused by PRV infection.

## 2. Materials and Methods

### 2.1. Viruses, Animals, and Cells

The PRV-GXLB-2013 strain was provided by the Laboratory of Preventive Veterinary Medicine, College of Animal Science and Technology, Guangxi University. The virus was propagated in PK-15 cells cultured in Dulbecco’s Modified Eagle Medium ( DMEM; Gibco, Thermo Fisher Scientific, Shanghai, China) supplemented with 10% fetal bovine serum (FBS, Baidi Science and Technology, Hangzhou, China) at 37 °C under 5% CO_2_ until cytopathic effects (CPE) reached approximately 80%. Viral titers were determined to be 10^7.2^ TCID_50_/mL using the Reed–Muench method [21]. Aliquots of the viral stock were stored at −80 °C for subsequent experiments.

The 5-week-old specific pathogen-free (SPF) Kunming mice (20 ± 2 g body weight, equal gender distribution) were obtained from Changsha Tianqin Biotechnology Co., Ltd. (Changsha, China) (Production License: SCXK [Xiang] 2022-0011). Mice were housed in a pathogen-free facility with ad libitum access to food and water. All animal procedures complied with the Institutional Animal Care and Use Committee of Guangxi University (Ethics Approval No.: GXU-2022-210).

Mouse astrocyte-derived C8-D1A cells were generously provided by the Department of Infectious Diseases, College of Animal Science and Technology, Guangxi University. Cells were maintained in DMEM supplemented with 10% FBS and a 1% penicillin–streptomycin–amphotericin B antibiotic cocktail (Baidi Science and Technology, Hangzhou, China) and cultured at 37 °C in 5% CO_2_.

### 2.2. Drugs and Chemicals

Necrosulfonamide (NSA) was obtained from Selleck Chemicals (Shanghai, China). Anti-pseudorabies virus antibody (ab3534) was acquired from Abcam (Cambridge, UK). Serum nitric oxide (NO), total nitric oxide synthase (TNOS), inducible NOS (iNOS), and constitutive NOS (cNOS) assay kits were purchased from Nanjing Jiancheng Bioengineering Institute (Nanjing, China). MLKL, TNF-α, IL-1β, IL-6, and IL-8 ELISA kits were provided by Enzyme Immunoassay Biotechnology Co. (Yancheng, China). Dimethyl sulfoxide (DMSO), an enhanced CCK-8 proliferation assay kit, SparkZol RNA isolation reagent, SPARKscript II All-in-One RT SuperMix for qPCR (with gDNA Eraser), and 2× SYBR Green qPCR Master Mix (with ROX) were purchased from Sparkjade Biotechnology Co., Ltd. (Qingdao, China).

### 2.3. Animal Studies

Following a 12 h fasting period, mice were randomly divided into four groups (n = 16 per group, 1:1 male-to-female ratio): blank control group, 10^2^ TCID_50_ PRV group, 10^3^ TCID_50_ PRV group, and 10^4^ TCID_50_ PRV group. All groups received intramuscular injections (100 μL) of either DMEM culture medium or PRV-GXLB-2013 viral suspension at the specified titers. Animals were maintained under standard housing conditions with ad libitum access to sterilized feed and water. Mice were immediately anesthetized via intraperitoneal injection of 1% sodium pentobarbital (30 mg/kg) following retro-orbital blood collection, then euthanized and subjected to aseptic necropsy. Surviving mice were euthanized on day 7 post-infection. Serum samples were collected from all animals for subsequent analysis.

### 2.4. Observation of Clinical Symptoms

Following PRV-GXLB-2013 infection, mice were monitored daily for clinical signs, including mental status, food intake, and locomotor activity. The onset, duration, and progression of neurological symptoms were systematically recorded, along with morbidity and mortality rates. Neurological function was assessed daily for seven days post-infection using a modified Neurological Severity Score (mNSS) system. This comprehensive behavioral assessment evaluates motor function, sensory responses, reflex integrity, and balance capabilities, with total scores ranging from 0 (normal) to 18 (maximal neurological impairment). Higher scores correlate with more severe neurological dysfunction [21].

### 2.5. Detection of Blood–Brain Barrier Permeability by the Evans Blue Method

Three randomly selected mice per group received tail vein injections of 2% Evans Blue (EB; 5 mL/kg). Dye extravasation was monitored by observing bluish discoloration of extremities and auricles. Two hours post-injection, mice were anesthetized and subjected to transcardial perfusion with saline. Brains were harvested and wet weights were recorded. Brain tissues were homogenized in formamide (100 mg tissue/mL) and incubated at 45 °C for 24 h. Following centrifugation (1000× *g*, 10 min), EB absorbance in the supernatant was measured at 610 nm. The EB content in brain tissue was calculated as: EB content (μg/g tissue) = [A610 × EB standard curve concentration (μg/mL)] × formamide volume (mL)/brain wet weight (g).

### 2.6. Determination of Water Content in the Brain Tissue

Brain tissue was taken from three mice per group and dissected into cerebral hemispheres, cerebellum, and brainstem. Following saline rinsing and surface moisture removal with filter paper, tissues were precisely weighed to obtain wet mass (W1). Samples were then dehydrated in a 105 °C drying oven for 48 h and reweighed to determine dry mass (W2). The water content in the brain tissue was calculated using the formula: [(W1 − W2)/W1] × 100%.

### 2.7. Histopathologic Observation of Brain Tissue

Brain tissues were fixed in 4% paraformaldehyde for 24 h, followed by thorough rinsing in running tap water. Tissues were then dehydrated through a graded ethanol series, cleared in xylene, and embedded in paraffin. Serial sections (5 µm thickness) were cut using a microtome and mounted on slides. After standard deparaffinization with xylene and rehydration through an ethanol gradient, tissue sections were processed for histological analysis. Hematoxylin and eosin (H&E) staining was performed to assess general neuropathological alterations. Additionally, Nissl staining was conducted to evaluate neuronal morphology and integrity.

### 2.8. Detection of Viral Particle Distribution in Brain Tissue by Immunohistochemical Staining

Immunohistochemical (IHC) staining was performed to detect viral particles using an anti-PRV monoclonal antibody. Brain tissue paraffin sections were deparaffinized in xylene and rehydrated through a graded ethanol series. Antigen retrieval was conducted by microwave heating (medium power, 10 min) in EDTA buffer. Endogenous peroxidase activity was quenched by a 15 min incubation with 3% hydrogen peroxide. Non-specific binding sites were blocked with rapid containment solution for 30 min at room temperature. Sections were then incubated overnight at 4 °C with rabbit anti-PRV polyclonal antibody (1:100 dilution; Abcam, ab3534, Cambridge, UK), followed by three PBS washes. The secondary antibody, goat anti-rabbit IgG H&L (HRP; Abcam, ab205718, Cambridge, UK), was applied for 20 min at room temperature. After additional PBS washes, color development was performed using DAB substrate until optimal brown staining appeared and then immediately terminated by distilled water rinses. Finally, sections were counterstained with hematoxylin, dehydrated, and mounted for microscopic examination of PRV particle distribution in hippocampal regions.

### 2.9. Detection of Viral Load in Brain Tissue by Real-Time PCR

PRV genomic DNA was isolated from brain tissue using the SPARKeasy Virus Genomic DNA Extraction Kit (Qingdao, China). Viral DNA copy numbers were quantified by absolute qPCR using extracted DNA as a template on a LightCycler 96 real-time PCR system. The qPCR was performed using the 2× SYBR Green qPCR Master Mix (with ROX) according to the manufacturer’s instructions. The quantification was based on a standard curve generated using serial dilutions of PRV plasmid DNA containing the gE gene, with the following equation: Y = −3.520X + 39.436, where X represents the log_10_ copy number, and Y represents the Ct value [22]. The following primer set targeting the gE gene was used for amplification. These primers were previously described in [22] and were as follows:

gE-F: 5′-CGTGTTCTTTGTGGCGGTG-3′

gE-R: 5′-AGCGTGGCGGTAAAGTTCTC-3′

### 2.10. Detection of Inflammatory Factor Levels in Brain Tissue and Serum

Brain tissues were homogenized in ice-cold lysis buffer (1:9 *w*/*v* ratio) using mechanical disruption, followed by centrifugation (3000× *g*, 10 min, 4 °C), to obtain clarified supernatants. Subsequently, MLKL protein levels, proinflammatory cytokine concentrations (TNF-α, IL-1β, IL-6, and IL-8), nitric oxide (NO) content, and nitric oxide synthase activities (TNOS, iNOS, and cNOS) in brain homogenates were quantified using commercial assay kits according to manufacturers’ protocols. Serum levels of TNF-α, IL-1β, IL-6, and IL-8 were similarly determined using standardized ELISA procedures.

### 2.11. PRV Infection of C8-D1A Cells In Vitro

The in vitro effect of PRV on C8-D1A cell viability was assessed using CCK-8 assay. Cells were seeded in 96-well plates at 2 × 10^5^ cells/mL (100 μL/well) and cultured overnight at 37 °C with 5% CO_2_. The C8-D1A cell was infected with PRV-GXLB-2013 at MOI = 0, 0.001, 0.01, 0.1, 1, and 10, with six replicates per condition. Following 2 h viral adsorption at 37 °C, wells were washed thrice with PBS and maintained in 5% FBS-DMEM. At 6, 12, 24, and 48 h post-infection, 10 μL of CCK-8 reagent was added to each well and incubated for 1 h. Absorbance at 450 nm was measured, and cell viability was calculated as: [(OD_450_ sample − OD_450_ blank)/(OD_450_ control − OD_450_ blank)] × 100%.

C8-D1A cells were inoculated into 6-well plates at 1 × 10^6^ cells/mL (2 mL/well) and incubated at 37 °C with 5% CO_2_ until 80% confluence. According to the results of the effect of PRV-GXLB-2013 on the activity of C8-D1A cells, cells were infected with PRV at MOI = 0, 0.01, 0.1, and 1, respectively. Following viral adsorption for 2 h at 37 °C (with gentle agitation every 30 min), cells underwent three PBS washes before maintenance in 5% FBS-DMEM. Supernatant collection occurred at 6, 12, 24, and 48 h post-infection, with immediate storage at −80 °C. TNF-α and IL-6 concentrations were quantified using commercial ELISA kits according to manufacturer specifications, with all experimental procedures performed under standardized conditions to ensure reproducibility.

### 2.12. Real-Time PCR Assessment of mRNA Expression

Total RNA was isolated from brain tissues or C8-D1A cells using the TRIzol reagent. cDNA synthesis was performed with the SPARKscript II All-in-One RT SuperMix (with gDNA Eraser), and the resulting cDNA was stored at −20 °C. Quantitative PCR was conducted using the 2× SYBR Green qPCR Master Mix (with ROX) on a LightCycler 96 real-time PCR system under the following cycling conditions: initial denaturation at 94 °C for 3 min; 40 cycles of 94 °C for 10 sec; and 60 °C for 30 sec. Amplification curves and melt curves were analyzed to determine Ct values. Relative gene expression was calculated using the 2^−ΔΔCt^ method, with β-actin as the endogenous control. Primers were designed using NCBI resources and commercially synthesized by GeneScript Biotechnology Co., Ltd. (Nanning, China). Primer sequences are shown in Table 1.

### 2.13. Evaluation of NSA Intervention in PRV-GXLB-2013 Infection Using Both In Vivo (Mouse Model) and In Vitro (C8-D1A Astrocyte Culture) Systems

Mice received intraperitoneal injections of either the NSA inhibitor (20 mg/kg in 100 μL saline) or the vehicle control (100 μL saline) 1 h prior to viral inoculation. On day 7 post-infection, animals were anesthetized and euthanized for brain tissue collection.

C8-D1A cultures were pretreated with 10 μM NSA (DMSO-solubilized) 30 min prior to infection. Following 12 h incubation, cells were monitored for cytopathic effects (CPE) using phase-contrast microscopy. Both culture supernatants and cellular fractions were harvested for subsequent analysis. Meanwhile, at 12 h post-inoculation, the supernatants were aspirated, and cells were gently washed once with PBS. Cells were then stained with Hoechst 33342 (nuclear dye; blue fluorescence)/propidium iodide (PI; necrosis marker; red fluorescence) working solution (Solarbio, Beijing, China) and incubated at 4 °C for 30 min in the dark. Fluorescence microscopy was performed to visualize cellular staining patterns: viable cells displayed uniform blue nuclear staining, whereas necrotic cells exhibited both intense red cytoplasmic and blue nuclear fluorescence.

Brain tissue samples and cellular specimens were trimmed into 1 mm^3^ fragments and fixed in 2.5% glutaraldehyde, followed by post-fixation in 1% osmium tetroxide. Samples were dehydrated through a graded acetone series, embedded in epoxy resin, and sectioned ultrathin (70–90 nm). Sections were double-stained with uranyl acetate and lead citrate before examination using transmission electron microscopy (TEM).

### 2.14. Statistical Analysis

All data were analyzed using IBM SPSS Statistics (version 27.0) and were presented as mean ± standard error of the mean (SEM). After verifying normality and homogeneity of variance assumptions, one-way analysis of variance (ANOVA) was performed, followed by Duncan’s post hoc multiple comparisons test. Statistically significant differences (*p* < 0.05) between treatment groups were indicated by distinct lowercase letters, while highly significant differences (*p* < 0.01) were denoted by distinct uppercase letters. All figures were generated using GraphPad Prism (version 9.5).

## 3. Results

### 3.1. PRV-GXLB-2013 Exhibited Neuroinvasive Properties in Mice, Resulting in Significant Neuropathological Alterations

**Figure 1:**
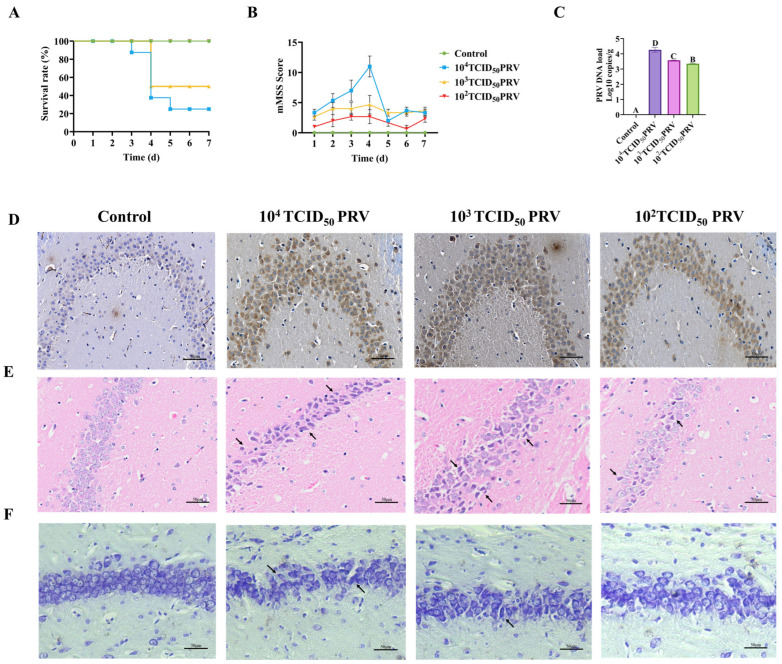
PRV-GXLB-2013 exhibited neuroinvasive properties in mice, resulting in significant neuropathological alterations in the nervous system. Mortality was monitored for 7 days post-infection (dpi), with daily neurological assessments performed using a standardized scoring system. Brain tissues were collected at 7 dpi for subsequent analyses. (**A**) Survival curves following PRV-GXLB-2013 infection. (**B**) neurological symptom progression, showing dose-dependent CNS impairment. The 10^4^ TCID_50_ group exhibited the most severe symptoms, followed by 10^3^ TCID_50_, while 10^2^ TCID_50_ showed no significant clinical manifestations. (**C**) Quantitative viral load in brain tissue by qPCR; (**D**) immunohistochemical detection of PRV antigens in hippocampal regions. Between groups, different uppercase letters indicate extremely significant differences (*p* < 0.01), and the same letter markings indicate no significant differences (*p* > 0.05). (**E**) hippocampal histopathology (H&E staining), with arrows indicating focal neuronal degeneration. (**F**) Nissl staining revealing neuronal abnormalities (arrows: disrupted Nissl bodies).

As shown in Figure 1A,B, mice infected with 10^4^ TCID_50_ PRV developed severe neurological manifestations 48 h post-infection, including lethargy, anorexia, dyspnea, pruritus, and localized alopecia, with ulcerative bleeding at the inoculation site. This group exhibited an 80% mortality rate by day 5 post-infection. In the 10^3^ TCID_50_ PRV cohort, animals displayed moderate symptoms, including pruritus, self-excoriation, and dermal ulceration, with 50% mortality occurring by day 4. The 10^2^ TCID_50_ PRV-infected mice remained asymptomatic throughout the observation period, with 100% survival.

Viral DNA extracted from brain tissue was quantified through absolute quantitative PCR analysis in this study. As shown in Figure 1C, all PRV-infected groups exhibited significantly elevated viral copy numbers in brain tissue, compared to controls (*p* < 0.01). Consistent with these findings, immunohistochemical analysis revealed extensive viral antigen staining in PRV-GXLB-2013-infected brains (Figure 1D), predominantly localized to neuronal cytoplasm in hippocampal regions. The staining intensity showed a clear dose-dependent relationship, with the 10^4^ TCID_50_ group demonstrating the strongest immunoreactivity, correlating with the highest viral load. These results collectively demonstrated robust PRV replication and neurotropism in infected mice.

Histopathological analysis revealed distinct morphological alterations in hippocampal neurons across treatment groups (Figure 1E). Control group neurons exhibited normal cytoarchitecture, characterized by spherical nuclei with well-defined margins and densely packed, regularly arranged somata. In contrast, PRV-GXLB-2013 infection induced dose-dependent neuropathological changes. The 10^4^ TCID_50_ group showed severe neuronal disorganization, featuring cellular shrinkage, nuclear pyknosis, cytoplasmic vacuolization, and partial cytolysis. Neurons in the 10^3^ TCID_50_ group displayed moderate pathology, including irregular cellular distribution, eosinophilic condensation, and nuclear deformation. The 10^2^ TCID_50_ group exhibited mild neurodegeneration, with reduced neuronal density, dispersed arrangement, and evidence of nuclear lysis.

Histological examination revealed a distinct neuronal pathology across treatment groups (Figure 1F). Control group hippocampi exhibited numerous neurons with intact morphologies, well-defined cytoplasmic boundaries, compact organization, and uniformly distributed Nissl substance, showing characteristic basophilic staining. In contrast, the 10^4^ TCID_50_ PRV-infected group displayed marked neuronal disorganization, featuring (i) reduced Nissl body density, (ii) chromatolysis (evidenced by decreased basophilia), and (iii) structural deformation of remaining Nissl bodies. The 10^3^ TCID_50_ group showed irregular neuronal distribution, with a heterogeneous Nissl staining intensity. The 10^2^ TCID_50_ group demonstrated sparse neuronal arrangement accompanied by significant Nissl body depletion and hypochromasia.

### 3.2. PRV-GXLB-2013 Infection Produced Damage to the Blood–Brain Barrier

**Figure 2:**
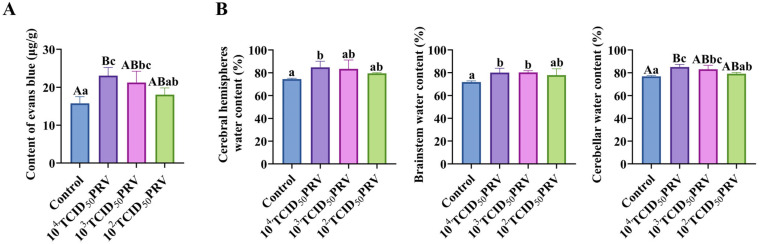
PRV-GXLB-2013 infection disrupted the BBB. (**A**) PRV-GXLB-2013-induced BBB dysfunction in mice. (**B**) regional brain edema development post-PRV-GXLB-2013 infection. Between groups, different uppercase letters indicate extremely significant differences (*p* < 0.01), different lowercase letters indicate significant differences (*p* < 0.05), and the same letter markings indicate no significant differences (*p* > 0.05).

EB extravasation assays were performed to quantitatively assess PRV-GXLB-2013-induced blood–brain barrier (BBB) disruption across varying viral doses. Figure 2A demonstrates a dose-dependent BBB compromise, with the 10^4^ TCID_50_ group exhibiting markedly elevated EB content (*p* < 0.01) and significantly increased BBB permeability (*p* < 0.01), relative to controls. The 10^3^ TCID_50_ group showed moderate but significant increases in both parameters (*p* < 0.05). A concurrent assessment of brain water content revealed pronounced neurovascular dysfunction (Figure 2B). The 10^4^ TCID_50_ group displayed (i) highly significant cerebellar edema (*p* < 0.01) and (ii) significant fluid accumulation in cerebral hemispheres and the brainstem (*p* < 0.05). The 10^3^ TCID_50_ group showed selective edema in the brainstem and cerebellar regions (*p* < 0.05).

### 3.3. PRV-GXLB-2013 Infection Triggered Significant Upregulation of Proinflammatory Mediators in Both Brain Tissue and Serum

**Figure 3:**
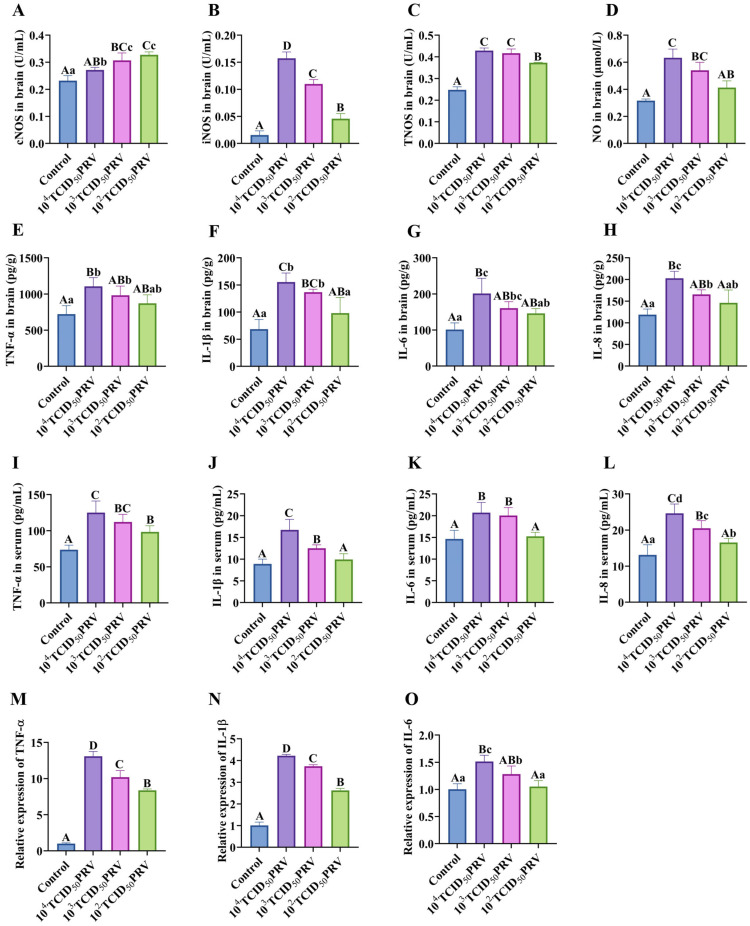
PRV-GXLB-2013-induced inflammatory response in the nervous system. (**A**–**H**) cNOS, iNOS, and TNOS activities, as well as NO, TNF-α, IL-1β, IL-6, and IL-8 levels, were determined by the kit in mouse brain tissue. (**I**–**L**) Determination of TNF-α, IL-1β, IL-6, and IL-8 levels in mouse serum by appropriate ELISA kits. (**M**–**O**) Detection of TNF-α, IL-1β, and IL-6 mRNA levels in mouse brain tissue by RT-qPCR. Between groups, different uppercase letters indicate extremely significant differences (*p* < 0.01), different lowercase letters indicate significant differences (*p* < 0.05), and the same letter markings indicate no significant differences (*p* > 0.05).

PRV infection elicited a robust systemic inflammatory response, characterized by the dose-dependent upregulation of proinflammatory mediators across multiple biological compartments. Quantitative analyses revealed, when compared with the control group, significant elevations in brain tissue nitric oxide synthase (NOS) activity (cNOS, iNOS, and TNOS) and nitric oxide (NO) production (10^3^ TCID_50_ PRV: *p* < 0.01; 10^2^ TCID_50_ PRV and 10^4^ TCID_50_ PRV: *p* < 0.01 or *p* < 0.05), paralleled by increased cytokine levels (TNF-α, IL-1β, IL-6, and IL-8) in both brain tissue serum (ELISA) and brain tissue (RT-qPCR). Notably, the 10^4^ TCID_50_ PRV and 10^3^ TCID_50_ PRV groups exhibited pronounced neuroinflammation, with highly significant upregulations of TNF-α, IL-1β, and IL-6 mRNA (*p* < 0.01) and contents (*p* < 0.01 or *p* < 0.05), demonstrating a viral titer-dependent cytokine secretion gradient in the nervous system (Figure 3).

### 3.4. PRV-GXLB-2013 Infection of C8-D1A Cells Triggered Significant Upregulation of Proinflammatory Markers

**Figure 4:**
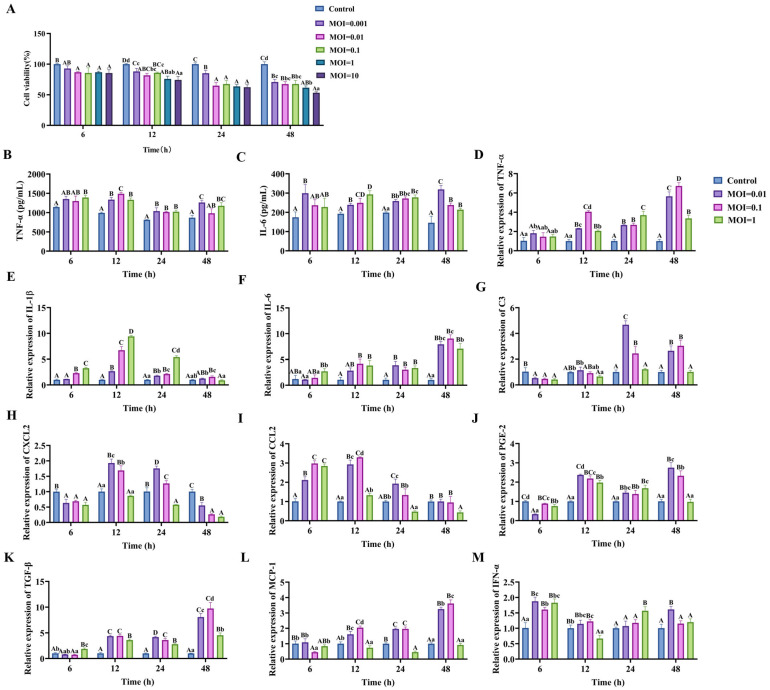
PRV-GXLB-2013 infection of C8-D1A cells triggered the upregulation of proinflammatory markers. (**A**) PRV-GXLB-2013 with different viral titers infected C8-D1A cells at different times, and the cell viability of C8-D1A cells was determined by CCK-8 assay. (**B**–**C**) PRV-GXLB-2013 with different viral titers infected C8-D1A cells at different times, and the levels of TNF-α and IL-6 in C8-D1A cells were measured by the corresponding ELISA kits. (**D**–**M**) PRV-GXLB-2013 with different viral titers infected C8-D1A cells at different times, and TNF-α, IL-1β, IL-6, C3, CXCL2, CCL2, PGE-2, TGF-β, MCP-1, and IFN-α mRNA levels were detected by RT-qPCR in C8-D1A cells. Between groups, different uppercase letters indicate extremely significant differences (*p* < 0.01), different lowercase letters indicate significant differences (*p* < 0.05), and the same letter markings indicate no significant differences (*p* > 0.05).

Infection of C8-D1A cells with PRV-GXLB-2013 resulted in a dose- and time-dependent reduction in cell viability, as quantified by CCK-8 assay, when compared with the control group, with highly significant decreases observed at MOI = 0.01 and MOI = 1 across all timepoints (6–48 h; *p* < 0.01) and at MOI = 0.1 for 6 h, 24 h, and 48 h (*p* < 0.01). Subsequent analysis of inflammatory mediators revealed a marked upregulation of IL-6 and TNF-α protein levels (ELISA; 12–24 h; *p* < 0.01) and the transcriptional activation of proinflammatory genes (RT-qPCR; 12 h; MOI = 0.1), including TNF-α, CCL2, IL-1β, IL-6, CXCL2, MCP-1, IFN-α, PGE-2, and TGF-β (*p* < 0.01) (Figure 4). Based on these findings, an MOI of 0.1 and 12 h post-infection were established as standardized parameters for subsequent experiments, optimally capturing the virus-induced inflammatory cascade while maintaining cell viability for mechanistic studies.

### 3.5. PRV-GXLB-2013 Infection Robustly Activated the NF-κB/MLKL Signaling Axis in Both In Vivo Murine Models and Ex Vivo Astrocyte Cultures

**Figure 5:**
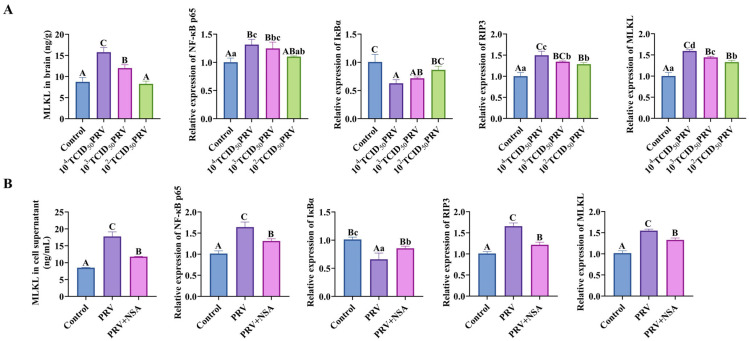
PRV-GXLB-2013 infection in vivo and ex vivo activated the NF-κB/MLKL signaling pathway. (**A**) PRV-GXLB-2013-infected mice with different viral titers were assayed for MLKL levels in mouse brain tissues by the corresponding ELISA kits, and RT-qPCR detected NF-κB p65, IκBα, RIP3, and MLKL mRNA levels in mouse brain tissues. (**B**) After the PRV-GXLB-2013 infection of C8-D1A cells, the levels of MLKL in the supernatant of C8-D1A cells were measured by the corresponding ELISA kits, and the levels of NF-κB p65, IκBα, RIP3, and MLKL mRNA were detected by RT-qPCR in C8-D1A cells. Between groups, different uppercase letters indicate extremely significant differences (*p* < 0.01), different lowercase letters indicate significant differences (*p* < 0.05), and the same letter markings indicate no significant differences (*p* > 0.05).

As shown in Figure 5, compared with the control group, the MLKL content of the brain tissues and C8-D1A cells of mice in the 10^4^ TCID_50_ PRV group and 10^3^ TCID_50_ PRV group were all highly elevated (*p* < 0.01). Compared with control, the relative mRNA expressions of NF-κB p65, RIP3, and MLKL genes in the brain tissues and C8-D1A cells of mice in the 10^4^ TCID_50_ PRV group and 10^3^ TCID_50_ PRV group were highly significantly or significantly increased (*p* < 0.01 or *p* < 0.05); the relative mRNA expression of IκBα genes in the 10^4^ TCID_50_ PRV group, 10^3^ TCID_50_ PRV group, and 10^3^ TCID_50_ PRV group mice showed a highly significant (*p* < 0.01) decrease in the relative mRNA expression of the IκBα gene in brain tissue and C8-D1A cells. This suggested that PRV-GXLB-2013 ex vivo infection activated the NF-κB/MLKL signaling pathway.

### 3.6. NSA Attenuated PRV-GXLB-2013-Induced Neuroinflammatory Damage in Both Murine Models and Cultured Astrocytes

**Figure 6:**
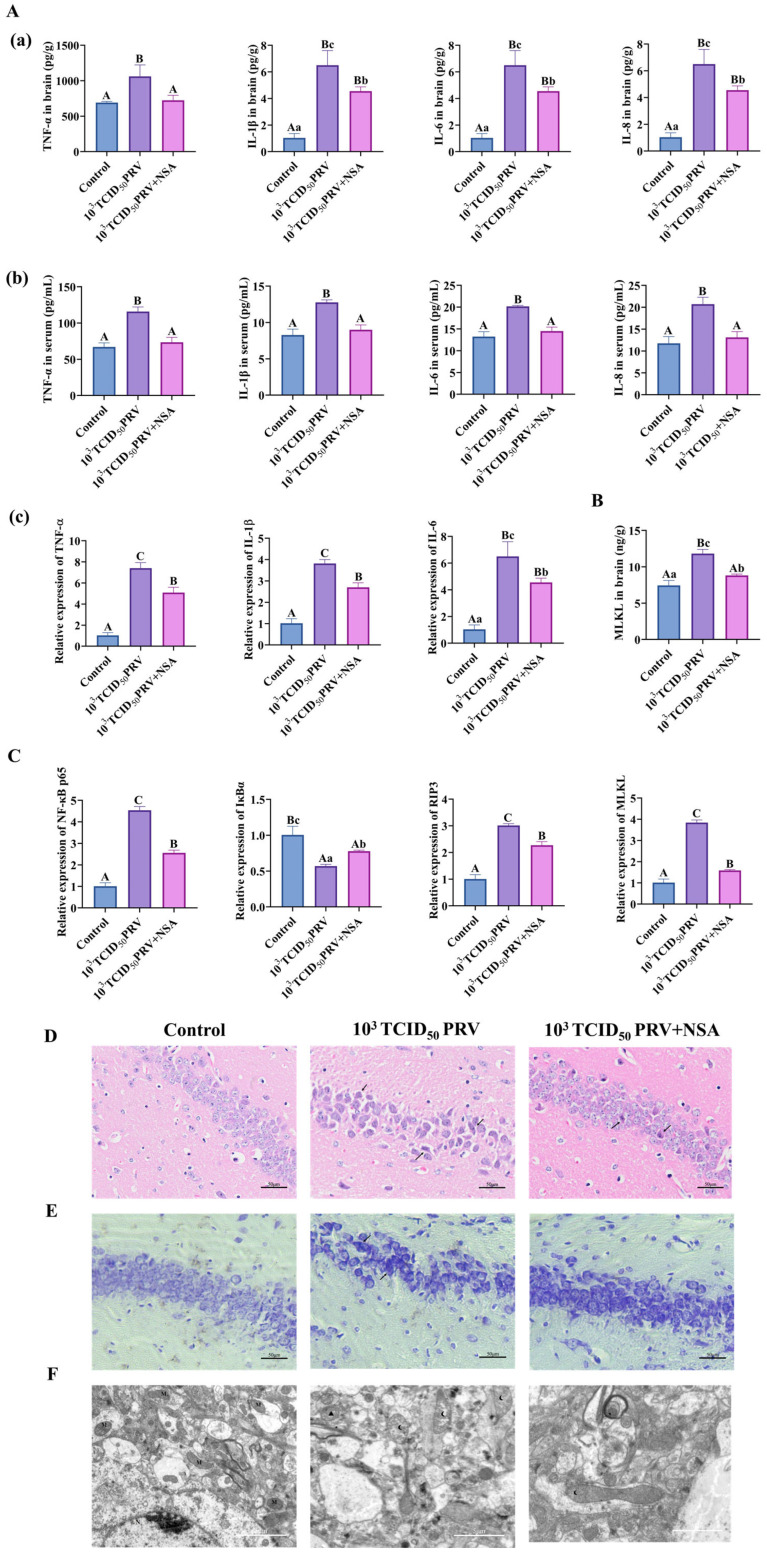
NSA could inhibit PRV-GXLB-2013-induced necrotic apoptosis in vivo. Treatment group mice received intraperitoneal NSA administration (20 mg/kg) 1 h prior to viral inoculation. On day 7 post-infection, animals were humanely euthanized under deep anesthesia (sodium pentobarbital, 50 mg/kg), followed by immediate brain tissue collection. Harvested tissues were snap-frozen in liquid nitrogen and stored at −80 °C for subsequent analysis. (**A**)—(**a**) Determination of TNF-α, IL-1β, IL-6, and IL-8 levels in mouse brain tissue by appropriate ELISA kits. (**b**) determination of TNF-α, IL-1β, IL-6, and IL-8 levels in mouse serum by appropriate ELISA kits. (**c**) detection of TNF-α, IL-1β, and IL-6 mRNA levels in mouse brain tissue by RT-qPCR. (**B**) Determination of MLKL levels in mouse brain tissue by appropriate ELISA kits. (**C**) Detection of NF-κB p65, IκBα, RIP3, and MLKL mRNA in mouse brain tissue by RT-qPCR. (**D**) HE staining reflecting lesions in the hippocampus of mice, with areas of neuronal damage shown by black arrows. (**E**) Nissl staining reflecting lesions in the hippocampus of mice, with abnormal Nissl vesicles shown by black arrowheads. (**F**) Transmission electron micrograph reflecting mitochondrial damage in the hippocampus of mouse brain tissue. M indicates mitochondria, N indicates nucleus, black pentagrams indicate swollen mitochondria, black triangles indicate solidified mitochondria, black crescents indicate structurally damaged mitochondria, and black arrowheads indicate disrupted mitochondrial membranes. Between groups, different uppercase letters indicate extremely significant differences (*p* < 0.01), different lowercase letters indicate significant differences (*p* < 0.05), and the same letter markings indicate no significant differences (*p* > 0.05).

**Figure 7:**
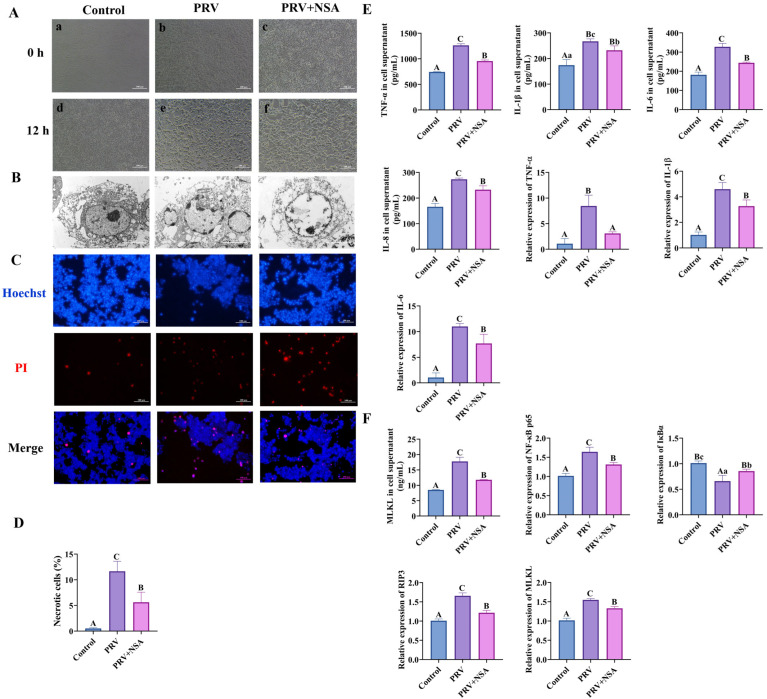
NSA inhibits PRV-GXLB-2013-induced necroptosis in vitro. C8-D1A cells were infected with PRV-GXLB-2013 (MOI = 0.1) for 12 h to establish baseline pathology prior to NSA treatment. Cells were pretreated with NSA (10 μM) 30 min prior to viral exposure. after which they were cultured for 12 h after the virus reception to observe the cytopathic effect (CPE), and the cell supernatant and cell samples were collected and frozen at −80°C for storage. (**A**) Microscopic observation of CPE induced by PRV-GXLB-2013 infection in C8-D1A cells. (a,b,c) Cells in each group treated accordingly. (d,e,f) Cells in each group after 12 h of incubation. (**B**) Transmission electron micrographs reflecting the state of cell membrane damage. (**C**,**D**) Levels of inflammatory factors in C8-D1A cells were determined by ELISA, and their mRNA expression was analyzed by RT-qPCR. (**E**,**F**) MLKL protein levels in C8-D1A cells were measured by ELISA, and mRNA levels of NF-κB p65, IκBα, RIP3, and MLKL were analyzed by RT-qPCR. Between groups, different uppercase letters indicate extremely significant differences (*p* < 0.01), different lowercase letters indicate significant differences (*p* < 0.05), and the same letter markings indicate no significant differences (*p* > 0.05).

As shown in Figure 6A–C and Figure 7E–F, infection with 10^3^ TCID_50_ of PRV-GXLB-2013 resulted in significantly elevated levels of TNF-α, IL-1β, IL-6, IL-8, and MLKL in brain tissues, serum, and C8-D1A cells, compared to the control group (*p* < 0.01). Conversely, NSA treatment markedly reduced these inflammatory and necroptotic markers post-infection (*p* < 0.01). Additionally, PRV-GXLB-2013 infection induced a highly significant upregulation in mRNA expressions of TNF-α, IL-1β, IL-6, NF-κB p65, RIP3, and MLKL (*p* < 0.01), alongside a pronounced downregulation of IκBα (*p* < 0.01), in both brain tissues and C8-D1A cells. In contrast, NSA administration significantly attenuated the expressions of pro-inflammatory and necroptotic genes (*p* < 0.01 or *p* < 0.05) while restoring IκBα levels. These results suggest that PRV-GXLB-2013 triggers inflammatory and necroptotic responses via NF-κB signaling, which can be mitigated by NSA treatment.

Histopathological analysis revealed distinct neuronal alterations following PRV-GXLB-2013 infection (10^3^ TCID_50_), characterized by irregular neuronal distribution, cellular shrinkage, and nuclear condensation (Figure 6D). In contrast, NSA treatment significantly ameliorated these pathological changes, resulting in a more regular neuronal arrangement and reduced morphological abnormalities, although minor irregularities persisted. Further examination (Figure 6E) demonstrated that infected neurons exhibited uneven staining of Nissl bodies, compared to controls, whereas NSA administration restored more compact neuronal organization and produced uniformly darker Nissl staining. These findings collectively indicate that NSA treatment effectively mitigates PRV-induced neuronal damage, suggesting its potential neuroprotective role against viral pathogenesis.

This study evaluated the neuroprotective effects of NSA against PRV-GXLB-2013-induced mitochondrial damage through the ultrastructural analysis of hippocampal neurons using transmission electron microscopy (TEM) (Figure 6F). Comparative analysis revealed intact mitochondrial architecture in control neurons, with numerous organelles distributed throughout the cytoplasm. In contrast, PRV-infected neurons (10^3^ TCID_50_) exhibited severe mitochondrial pathology, including cristae disintegration, fragmentation, and occasional autophagic sequestration. Notably, NSA treatment substantially preserved mitochondrial integrity, with only minor cristae disruptions observed and overall organelle morphology maintained, demonstrating its protective capacity against viral-induced mitochondrial damage.

Electron microscopy revealed distinct cytopathic effects (CPE) in PRV-GXLB-2013-infected C8-D1A cells as early as 12 h post-infection (Figure 7A), characterized by cellular deformation, membrane shrinkage, and necrotic morphology. Ultrastructural analysis (Figure 7B) demonstrated hallmark features of viral-induced necrosis, including cellular swelling, nuclear chromatin dissolution, plasma membrane rupture, and cytoplasmic leakage. Notably, NSA treatment attenuated these pathological alterations, with the observable preservation of cellular architecture and reduced necrotic features, suggesting a protective effect against PRV-mediated cytolysis.

Hoechst 33342/propidium iodide (PI) dual staining was conducted on PRV-GXLB-2013-infected C8-D1A cells to characterize cell death mechanisms further. Quantitative analysis revealed a significant increase in both apoptotic and necrotic cell populations following infection, compared to controls (*p* < 0.01, Figure 7C,D). Notably, NSA treatment markedly reduced the proportion of necrotic cells relative to the infected group (*p* < 0.01), demonstrating its protective effect against PRV-induced cytopathic effects.

## 4. Discussion

PRV, a member of the Alphaherpesvirinae subfamily, is a neurotropic pathogen that causes fatal neurological disease in neonatal pigs, characterized by locomotor ataxia, paralysis, convulsions, and intense pruritus, with near 100% mortality [23,24]. PRV also exhibits broad zoonotic potential, infecting various mammalian species, including ruminants, carnivores, and rodents, often resulting in severe CNS dysfunction [24]. In our study, infection of mice with the PRV-GXLB-2013 strain recapitulated key features of PRV-induced neuropathogenicity, including rapid-onset neurological symptoms, high mortality rates, and extensive CNS damage. The dose of 10^3^ TCID_50_ was identified as optimal for inducing consistent neuroinflammation without immediate lethality, making it suitable for mechanistic studies.

PRV infection triggered a robust neuroinflammatory response, marked by BBB disruption, cerebral edema, and hemorrhage—hallmark features of viral encephalitis [25,26,27]. BBB dysfunction, assessed through Evans Blue extravasation and wet/dry weight analysis, showed a clear dose-dependent increase in vascular permeability, suggesting that PRV directly compromises BBB integrity through both cytotoxic edema and vascular alterations. These findings align with previous reports on PRV-induced CNS pathology and highlight the importance of BBB integrity in limiting viral neuroinvasion [26]. Consistent with its neurotropism, PRV exhibited strong replication capacity within the CNS, as evidenced by elevated viral genomic loads and antigen expression in brain tissues [25,28]. This neuroinvasive behavior likely reflects PRV’s utilization of neural entry routes, such as the trigeminal nerve, to access the CNS [29]. Histopathological analyses further revealed progressive, dose-dependent neurodegeneration, particularly in the hippocampus, with features such as neuronal atrophy, karyopyknosis, and laminal disorganization, consistent with established murine models of PRV infection [7,30].

Astrocytes, as the predominant glial cell type in the CNS, play critical roles in maintaining homeostasis and BBB function [31]. Upon PRV infection, astrocytes were activated and contributed to the neuroinflammatory cascade by releasing proinflammatory mediators, such as TNF-α, IL-1β, IL-6, CCL2, and PGE-2, which are known to exacerbate neuronal injury [32,33,34]. In vitro experiments using the astrocyte-like cell line C8-D1A confirmed that PRV infection significantly upregulated these inflammatory factors in a multiplicity of infection (MOI)-dependent manner, with MOI = 0.1 for 12–24 h emerging as the most effective condition for inducing inflammation.

The NF-κB signaling pathway plays a central role in regulating neuroinflammatory responses by driving the transcription of cytokines, chemokines, and adhesion molecules [35]. Our data demonstrated robust activation of the canonical NF-κB pathway following PRV infection, as indicated by increased p65 expression, IκBα degradation, and downstream cytokine production (TNF-α, IL-1β, and IL-6). These findings are consistent with previous studies showing that PRV activates NF-κB to promote inflammatory gene expression in both neurons and glial cells [36,37,38]. Importantly, we found that PRV-induced NF-κB activation led to significant increases in nitric oxide (NO), TNOS, and iNOS levels, suggesting a link between inflammation and oxidative stress. This pattern resembles a “cytokine storm” commonly observed in severe viral infections, underscoring the pathological relevance of sustained NF-κB activation during PRV-induced neuroinflammation [39].

Furthermore, our findings suggest that PRV-induced TNF-α release may serve as a key upstream signal for necroptosis induction. TNF-α has been shown to activate RIPK3, which phosphorylates MLKL to execute necroptosis [40,41,42,43,44,45,46]. Supporting this mechanism, PRV infection of both mouse brain tissue and C8-D1A cells resulted in elevated expressions of TNF-α, RIP3, and MLKL, indicating the activation of the necroptotic pathway. These observations align with previous reports demonstrating that herpesvirus (e.g., HSV-1) infection triggers RIP3/MLKL-mediated cell death in the CNS [47,48].

Notably, the pharmacological inhibition of necroptosis using NSA, an MLKL inhibitor, significantly attenuated the expression of inflammatory mediators (TNF-α, IL-1β, IL-6, NF-κB p65, RIP3, and MLKL) and restored IκBα levels, suggesting that necroptosis contributes substantially to PRV-induced neuroinflammation. Ultrastructural analysis via TEM further supported these findings, revealing mitochondrial swelling, cristae disruption, and plasma membrane damage in PRV-infected cells—all of which were alleviated by NSA treatment.

Taken together, our data indicate that PRV activates the NF-κB signaling pathway, leading to TNF-α production, which subsequently triggers RIP3/MLKL-mediated necroptosis and drives neuroinflammatory pathology.

## 5. Conclusions

PRV-GXLB-2013 infection induces necroptosis in the nervous system, triggering robust neuroinflammation in vivo and in vitro. Nonetheless, the precise molecular mechanisms underlying PRV-induced neuroinflammatory pathogenesis remain to be fully elucidated. These findings establish a critical foundation for developing targeted therapeutic strategies to mitigate PRV-induced neurological damage.

## Figures and Tables

**Table 1:** The sequence of primers for RT-qPCR.

Target Gene	Sequence (5′–3′)	GenBank Accession No.
*β-actin*	F: 5′-GCTCTGGCTCCTAGCACCAT-3′	NM_007393.5
	R: 5′-GCCACCGATCCACACAGAGT-3′	
*TNF-α*	F: 5′-CGCTCTTCTGTCTACTGAACTTCGG-3′	NM_001278601.1
	R: 5′-GTGGTTTGTGAGTGTGAGGGTCTG-3′	
*IL-6*	F: 5′-CGGAGAGGAGACTTCACAGAG-3′	NM_001314054.1
	R: 5′-CATTTCCACGATTTCCCAGA-3′	
*IL-1β*	F: 5′-CACTACAGGCTCCGAGATGAACAAC-3′	XM_006498795.5
	R: 5′-TGTCGTTGCTTGGTTCTCCTTGTAC-3′	
*IκBα*	F: 5′-CAGCAGCTCACCGAGGAC-3′	NM_010907.2
	R: 5′-AAAGCCAGGTCTCCCTTCAC-3′	
*NF-κB p65*	F: 5′-GACCTGGAGCAAGCCATTAG-3′	XM_006531694.4
	R: 5′-CGCACTGTCACCTGGAAGC-3′	
*RIP3*	F: 5′-CGGCTCTCGTCTTCAACAACTG-3′	NM_001164107.1
	R: 5′-CGAACTGTGCTTGGTCATACTTGG-3′	
*MLKL*	F: 5′-GTTTGTGAGTGTGGGCAATGATAAG-3′	XM_006531443.5
	R: 5′-AGGATGCTGGCTGGCTGAC-3′	
*C3*	F: 5′-CCAGCTCCCCATTAGCTCTG-3′	NM_009778.3
	R: 5′-GCACTTGCCTCTTTAGGAAGTC-3′	
*CXCL2*	F: 5′-AACATCCAGAGCTTGAGTGTGACG-3′	NM_009140.2
	R: 5′-GGGCTTCAGGGTCAAGGCAAAC-3′	
*CCL2*	F: 5′-ATTAAAAACCTGGATCGGAAC-3′	NM_133783.2
	R: 5′-GCATTAGCTTCAGATTTACGGGT-3′	
*IFN-α*	F: 5′-TACTCAGCAGACCTTGAACCT-3′	NM_010503.2
	R: 5′-CAGTCTTGGCAGCAAGTTGAC-3′	
*PGE2*	F: 5′-TGGAGGTGAATCCCGTGAGA-3′	XM_036152883.1
	R: 5′-AAACTCGGTCACCTCCTTGC-3′	
*TGF-β*	F: 5′-TGCGCTTGCAGAGATTAAAA-3′	NM_011333.3
	R: 5′-CGTCAAAAGACAGCCACTCA-3′	
*MCP-1*	F: 5′-CCACTCACCTGCTGCTACTCAT-3′	NM_011333.3
	R: 5′-TGGTGATCCTCTTGTAGCTCTCC-3′	

## Data Availability

All data are available from the corresponding author by request.

## References

[1] Mettenleiter T.C. (1991). Molecular biology of pseudorabies (Aujeszky’s disease) virus. Comp. Immunol. Microbiol. Infect. Dis..

[2] Wang H.M., Qiao Y.Y., Cai B.Y., Tan J., Na L., Wang Y., Lu H., Tang Y.D. (2023). Genome editing of pseudorabies virus in the CRISPR/Cas9 era: A mini-review. Front. Vet. Sci..

[3] McCarthy K.M., Tank D.W., Enquist L.W., Britt W.J. (2009). Pseudorabies virus infection alters neuronal activity and connectivity in vitro. PLoS Pathog..

[4] Ferrara G., Pagnini U., Parisi A., Amoroso M.G., Fusco G., Iovane G., Montagnaro S. (2024). A pseudorabies outbreak in hunting dogs in Campania region (Italy): A case presentation and epidemiological survey. BMC Vet. Res..

[5] Ai J.-W., Weng S.-S., Cheng Q., Cui P., Li Y.-J., Wu H.-L., Zhu Y.-M., Xu B., Zhang W.-H. (2018). Human Endophthalmitis Caused By Pseudorabies Virus Infection, China, 2017. Emerg. Infect. Dis..

[6] Liu Q., Wang X., Xie C., Ding S., Yang H., Guo S., Li J., Qin L., Ban F., Wang D. (2021). A Novel Human Acute Encephalitis Caused by Pseudorabies Virus Variant Strain. Clin. Infect. Dis..

[7] Xu L., Wei J.-F., Zhao J., Xu S.-Y., Lee F.-Q., Nie M.-C., Xu Z.-W., Zhou Y.-C., Zhu L. (2022). The Immunity Protection of Central Nervous System Induced by Pseudorabies Virus DelgI/gE/TK in Mice. Front. Microbiol..

[8] Hou Y., Wang Y., Zhang Y., Yu H., Zhao Y., Yi A. (2022). Human Encephalitis Caused by Pseudorabies Virus in China: A Case Report and Systematic Review. Vector-Borne Zoonotic Dis..

[9] Verpoest S., Redant V., Cay A.B., Favoreel H., De Regge N. (2018). Reduced virulence of a pseudorabies virus isolate from wild boar origin in domestic pigs correlates with hampered visceral spread and age-dependent reduced neuroinvasive capacity. Virulence.

[10] Lai I.-H., Chang C.-D., Shih W.-L. (2019). Apoptosis Induction by Pseudorabies Virus via Oxidative Stress and Subsequent DNA Damage Signaling. Intervirology.

[11] Romero N., Favoreel H.W., Sandri-Goldin R.M. (2021). Pseudorabies Virus Infection Triggers NF-κB Activation via the DNA Damage Response but Actively Inhibits NF-κB-Dependent Gene Expression. J. Virol..

[12] Romero N., Van Waesberghe C., Favoreel H.W. (2020). Pseudorabies Virus Infection of Epithelial Cells Leads to Persistent but Aberrant Activation of the NF-kappaB Pathway, Inhibiting Hallmark NF-kappaB-Induced Proinflammatory Gene Expression. J. Virol..

[13] Li X., Chen S., Zhang L., Niu G., Zhang X., Yang L., Ji W., Ren L. (2022). Coinfection of Porcine Circovirus 2 and Pseudorabies Virus Enhances Immunosuppression and Inflammation through NF-kappaB, JAK/STAT, MAPK, and NLRP3 Pathways. Int. J. Mol. Sci..

[14] Woznicki J.A., Saini N., Flood P., Rajaram S., Lee C.M., Stamou P., Skowyra A., Bustamante-Garrido M., Regazzoni K., Crawford N. (2021). TNF-alpha synergises with IFN-gamma to induce caspase-8-JAK1/2-STAT1-dependent death of intestinal epithelial cells. Cell Death Dis..

[15] Fan H., Zhang K., Shan L., Kuang F., Chen K., Zhu K., Ma H., Ju G., Wang Y.-Z. (2016). Reactive astrocytes undergo M1 microglia/macrohpages-induced necroptosis in spinal cord injury. Mol. Neurodegener..

[16] Northington F.J., Chavez-Valdez R., Graham E.M., Razdan S., Gauda E.B., Martin L.J. (2011). Necrostatin decreases oxidative damage, inflammation, and injury after neonatal HI. J. Cereb. Blood Flow Metab..

[17] Oñate M., Catenaccio A., Salvadores N., Saquel C., Martinez A., Moreno-Gonzalez I., Gamez N., Soto P., Soto C., Hetz C. (2020). The necroptosis machinery mediates axonal degeneration in a model of Parkinson disease. Cell Death Differ..

[18] Leem Y.H., Kim D.Y., Park J.E., Kim H.S. (2023). Necrosulfonamide exerts neuroprotective effect by inhibiting necroptosis, neuroinflammation, and α-synuclein oligomerization in a subacute MPTP mouse model of Parkinson’ s disease. Sci. Rep..

[19] Nailwal H., Chan F.K. (2019). Necroptosis in anti-viral inflammation. Cell Death Differ..

[20] Yu X., He S. (2016). The interplay between human herpes simplex virus infection and the apoptosis and necroptosis cell death pathways. Virol. J..

[21] O’connor T.W., Collins D., Read A.J., Hick P.M., Kirkland P.D. (2025). A Standardised Method to Quantify the Infectious Titre of Rabbit Haemorrhagic Disease Virus. Viruses.

[22] Deng H., Deng Y., Song T., Pang L., Zhu S., Ren Z., Guo H., Xu Z., Zhu L., Geng Y. (2024). Evaluation of the activity and mechanisms of oregano essential oil against PRV in vivo and in vitro. Microb. Pathog..

[23] Salogni C., Lazzaro M., Giacomini E., Giovannini S., Zanoni M., Giuliani M., Ruggeri J., Pozzi P., Pasquali P., Boniotti M.B. (2016). Infectious agents identified in aborted swine fetuses in a high-density breeding area: A three-year study. J. Vet. Diagn. Investig..

[24] Liu Q., Kuang Y., Li Y., Guo H., Zhou C., Guo S., Tan C., Wu B., Chen H., Wang X. (2022). The Epidemiology and Variation in Pseudorabies Virus: A Continuing Challenge to Pigs and Humans. Viruses.

[25] Li L., Wang R., Hu H., Chen X., Yin Z., Liang X., He C., Yin L., Ye G., Zou Y. (2021). The antiviral activity of kaempferol against pseudorabies virus in mice. BMC Vet. Res..

[26] Zhao Z., Nelson A.R., Betsholtz C., Zlokovic B.V. (2015). Establishment and Dysfunction of the Blood-Brain Barrier. Cell.

[27] Zhang Y., Shu X., Song C., Wu Y., Cui K., Zhang X., Sun Y., Shen H., Wei Q., Li J. (2025). Astrocyte-derived MMP-9 is a key mediator of pseudorabies virus penetration of the blood-brain barrier and tight junction disruption. Vet. Res..

[28] Cai X., Wang Z., Li X., Zhang J., Ren Z., Shao Y., Xu Y., Zhu Y. (2023). Emodin as an Inhibitor of PRV Infection In Vitro and In Vivo. Molecules.

[29] Klopfleisch R., Teifke J.P., Fuchs W., Kopp M., Klupp B.G., Mettenleiter T.C. (2004). Influence of tegument proteins of pseudorabies virus on neuroinvasion and transneuronal spread in the nervous system of adult mice after intranasal inoculation. J. Virol..

[30] Chen X., Xue J., Zou J., Zhao X., Li L., Jia R., Zou Y., Wan H., Chen Y., Zhou X. (2023). Resveratrol alleviated neuroinflammation induced by pseudorabies virus infection through regulating microglial M1/M2 polarization. Biomed. Pharmacother..

[31] Sofroniew M.V., Vinters H.V. (2010). Astrocytes: Biology and pathology. Acta Neuropathol..

[32] Aston-Jones G., Card J.P. (2000). Use of pseudorabies virus to delineate multisynaptic circuits in brain: Opportunities and limitations. J. Neurosci. Methods.

[33] Clark I.C., Gutiérrez-Vázquez C., Wheeler M.A., Li Z., Rothhammer V., Linnerbauer M., Sanmarco L.M., Guo L., Blain M., Zandee S.E.J. (2021). Barcoded viral tracing of single-cell interactions in central nervous system inflammation. Science.

[34] Lu Y., He M., Zhang Y., Xu S., Zhang L., He Y., Chen C., Liu C., Pi H., Yu Z. (2014). Differential pro-inflammatory responses of astrocytes and microglia involve STAT3 activation in response to 1800 MHz radiofrequency fields. PLoS ONE.

[35] Lawrence T. (2009). The nuclear factor NF-kappaB pathway in inflammation. Cold Spring Harb. Perspect. Biol..

[36] Ren C.Z., Hu W.Y., Zhang J.W., Wei Y.Y., Yu M.L., Hu T.J. (2021). Establishment of inflammatory model induced by Pseudorabies virus infection in mice. J. Vet. Sci..

[37] Ye C., Huang Q., Jiang J., Li G., Xu D., Zeng Z., Peng L., Peng Y., Fang R. (2021). ATP-dependent activation of NLRP3 inflammasome in primary murine macrophages infected by pseudorabies virus. Vet. Microbiol..

[38] Shih R., Wang C., Yang C. (2015). NF-kappaB Signaling Pathways in Neurological Inflammation: A Mini Review. Front. Mol. Neurosci..

[39] Sun W., Liu S., Huang X., Yuan R., Yu J. (2021). Cytokine storms and pyroptosis are primarily responsible for the rapid death of mice infected with pseudorabies virus. R. Soc. Open Sci..

[40] Cho Y.S., Challa S., Moquin D., Genga R., Ray T.D., Guildford M., Chan F.K.-M. (2009). Phosphorylation-driven assembly of the RIP1-RIP3 complex regulates programmed necrosis and virus-induced inflammation. Cell.

[41] Harris K.G., Morosky S.A., Drummond C.G., Patel M., Kim C., Stolz D.B., Bergelson J.M., Cherry S., Coyne C.B. (2015). RIP3 Regulates Autophagy and Promotes Coxsackievirus B3 Infection of Intestinal Epithelial Cells. Cell Host Microbe.

[42] Xu C., Wu J., Wu Y., Ren Z., Yao Y., Chen G., Fang E.F., Noh J.H., Liu Y.U., Wei L. (2021). TNF-α-dependent neuronal necroptosis regulated in Alzheimer’s disease by coordination of RIPK1-p62 complex with autophagic UVRAG. Theranostics.

[43] Chadwick W., Magnus T., Martin B., Keselman A., Mattson M.P., Maudsley S. (2008). Targeting TNF-alpha receptors for neurotherapeutics. Trends Neurosci..

[44] Gou H., Bian Z., Cai R., Chu P., Song S., Li Y., Jiang Z., Zhang K., Yang D., Li C. (2021). RIPK3-Dependent Necroptosis Limits PRV Replication in PK-15 Cells. Front. Microbiol..

[45] Zhou H., Zhou M., Hu Y., Limpanon Y., Ma Y., Huang P., Dekumyoy P., Maleewong W., Lv Z. (2022). TNF-alpha Triggers RIP1/FADD/Caspase-8-Mediated Apoptosis of Astrocytes and RIP3/MLKL-Mediated Necroptosis of Neurons Induced by Angiostrongylus cantonensis Infection. Cell Mol. Neurobiol..

[46] Geng L., Gao W., Saiyin H., Li Y., Zeng Y., Zhang Z., Li X., Liu Z., Gao Q., An P. (2023). MLKL deficiency alleviates neuroinflammation and motor deficits in the α-synuclein transgenic mouse model of Parkinson’s disease. Mol. Neurodegener..

[47] Guo H., Kaiser W.J., Mocarski E.S. (2015). Manipulation of apoptosis and necroptosis signaling by herpesviruses. Med. Microbiol. Immunol..

[48] Shao H., Wu W., Wang P., Han T., Zhuang C. (2022). Role of Necroptosis in Central Nervous System Diseases. ACS Chem. Neurosci..

